# Dental Trauma and Quality of Life in a Paediatric Population (Up to 14 Years): A Bibliometric Analysis

**DOI:** 10.3390/healthcare14040475

**Published:** 2026-02-13

**Authors:** Bianca Núbia Souza-Silva, Danilo Cassiano Ferraz, Walbert de Andrade Vieira, João Marcos da Costa Ribeiro, Gabriel Phelipe de Paula Santos, Nathalia de Oliveira Domingos, Saul Martins Paiva, Carlos José Soares, Luiz Renato Paranhos

**Affiliations:** 1 Department of Dentistry, Universidade Federal de Sergipe, Aracaju 49060-108, SE, Brazil; bianubia93@gmail.com; 2 Department of Endodontics, School of Dental Medicine, University of Pittsburgh, Pittsburgh, PA 15213, USA; daniloferraz@pitt.edu; 3 Department of Dentistry, Centro Universitário das Faculdades Associadas (UNIFAE), São João da Boa Vista 13870-377, SP, Brazil; walbert.vieira18@gmail.com; 4 Postgraduate Program in Dentistry, Universidade Federal de Uberlândia, Uberlândia 38405-320, MG, Brazil; joaomarcosribeiro6725@gmail.com (J.M.d.C.R.); gsantos.dentistry@gmail.com (G.P.d.P.S.); nathalia.oliveira.d@hotmail.com (N.d.O.D.); 5 Department of Pediatric Dentistry, Faculty of Dentistry, Universidade Federal de Minas Gerais, Belo Horizonte 31270-901, MG, Brazil; smpaiva@uol.com.br; 6 Department of Operative Dentistry and Dental Materials, Universidade Federal de Uberlândia, Uberlândia 38405-320, MG, Brazil; carlosjsoares@ufu.br; 7 Department of Orthodontics, School of Dentistry, Universidade Federal de Uberlândia, Uberlândia 38405-320, MG, Brazil

**Keywords:** dental trauma, oral health-related quality of life, child, preschool child, adolescent, bibliometric analysis

## Abstract

**Background/Objectives**: Dental trauma is common in childhood and may negatively affect oral health-related quality of life (OHRQoL). Given the growing volume and diversity of publications on this topic, a bibliometric approach is suitable for mapping scientific production, collaboration patterns, thematic evolution, and citation dynamics. This study aimed to perform a bibliometric analysis of the literature addressing the impact of dental trauma on OHRQoL in a paediatric population up to 14 years of age. **Methods**: A bibliometric study was conducted using Clarivate’s Web of Science Core Collection (WoS-CC), selected for its standardized citation indexing and suitability for bibliometric analyses. Publications retrieved up to August 2025, without restrictions on language or year, were analyzed using VOSviewer (version 1.6.20) and Biblioshiny (Bibliometrix package). Indicators included scientific output, collaboration networks, keyword co-occurrence, thematic evolution, and citation performance. Spearman’s correlation was used to explore relationships between citation counts, journal impact factor, and year of publication. **Results**: A total of 107 articles published between 2006 and 2025 were included. Scientific output increased steadily, with publications concentrated in specific countries, notably Brazil and India. The predominant research focus concerned the impact of dental trauma on children’s quality of life. *Dental Traumatology* was the most productive journal and showed high local citation impact. Citation analysis demonstrated a weak positive correlation between citation counts and journal impact factor (rho = 0.37, *p* < 0.001) and a strong negative correlation with year of publication (rho = −0.84, *p* < 0.001). **Conclusions**: This bibliometric analysis identifies research trends, thematic stability, and collaboration patterns in studies on dental trauma and OHRQoL in children, highlighting regional concentration and limited international collaboration.

## 1. Introduction

Oral health-related quality of life (OHRQoL) is a multidimensional construct that reflects the influence of oral conditions on individuals’ physical, emotional, and social well-being [1]. In paediatric populations, OHRQoL has been widely used to capture the broader consequences of oral diseases beyond clinical signs, encompassing effects on daily activities, psychosocial development, and family dynamics [2,3,4]. As childhood represents a critical period of physical and emotional development, oral conditions occurring at this stage may produce impacts that extend beyond the immediate clinical context.

Among oral health problems affecting children, dental trauma stands out due to its high frequency and potential for long-term consequences. Traumatic dental injuries are prevalent across all age groups but occur disproportionately in childhood, particularly during early developmental stages [5,6]. Changes in lifestyle patterns, including increased participation in sports and recreational activities, have been associated with a rising incidence of dental trauma among children in recent decades [7,8]. Epidemiological evidence indicates substantial variability in prevalence according to geographic region, socioeconomic status, and demographic characteristics, with greater vulnerability observed among younger children and those living in less favorable social contexts [9,10].

Dental trauma may involve hard dental tissues and supporting structures, leading to pain, functional impairment, esthetic concerns, and psychological distress [11,12]. In primary dentition, these injuries are of particular concern because primary teeth play a fundamental role in speech development, mastication, and the maintenance of space for permanent teeth [13,14,15]. Consequently, traumatic injuries may negatively influence children’s oral health-related quality of life and indirectly affect family well-being, reinforcing the relevance of this condition from both clinical and public health perspectives.

Reflecting this relevance, the scientific literature addressing dental trauma and its impact on children’s OHRQoL has expanded considerably over time. Existing narrative reviews and systematic syntheses primarily focus on clinical outcomes or effect estimates, offering limited insight into patterns of scientific productivity, thematic development, collaboration networks, and knowledge dissemination. However, despite this growing body of evidence, the structure and dynamics of scientific production in this field remain insufficiently explored from a bibliometric perspective. There is a lack of quantitative assessments that systematically map publication trends, citation impact, collaborative networks, and thematic evolution over time.

Bibliometric analysis offers a methodological framework capable of addressing this gap by applying quantitative techniques to large bodies of scientific literature, enabling the evaluation of research productivity, academic influence, and knowledge development within a specific field [16,17,18]. Unlike systematic or scoping reviews, which focus on synthesizing clinical evidence, bibliometric studies provide a macroscopic perspective, allowing the identification of dominant research themes, underexplored domains, and structural characteristics of the literature.

Focusing on children up to 14 years of age allows the inclusion of preschool and school-aged populations, which represent critical developmental phases characterized by higher exposure to traumatic dental injuries and distinct psychosocial implications, while minimizing conceptual overlap with late adolescence. Therefore, no comprehensive bibliometric analysis has yet been conducted to characterize the scientific landscape of this research area. Therefore, this study aimed to perform a bibliometric analysis of the literature on the impact of dental trauma on OHRQoL in a paediatric population up to 14 years of age, assessing scientific productivity, citation performance, collaboration patterns, and thematic evolution.

## 2. Materials and Methods

### 2.1. Search Strategy

The search was conducted in August 2025 using Clarivate’s Web of Science Core Collection (WoS-CC). This database was selected due to its comprehensive coverage of dental journals and the availability of standardized citation and bibliometric indicators. No restrictions were applied regarding language or year of publication. This review followed the recommendations of the Guideline for Reporting Bibliometric Reviews of the Biomedical Literature (BIBLIO) [19,20].

To ensure comprehensive coverage, the search strategy was structured into three conceptual blocks encompassing traumatic dental injuries, the paediatric population, and associated biopsychosocial and educational variables (see Table 1). The final query employed the Boolean operator *AND* to combine these sets (#1 AND #2 AND #3). This approach was designed to capture the interplay between dental trauma and its broader consequences for well-being and academic outcomes within the paediatric population.

### 2.2. Time Period

The search covered all documents published from inception, with no starting date restriction, up to August 2025. This broad temporal scope was adopted to provide a comprehensive historical overview of the scientific literature on the subject.

### 2.3. Eligibility Criteria

The included articles assessed the impact of trauma on the paediatric population, defined as individuals aged 0 to 14 years (preschool children, children, and adolescents up to 14 years). This age range was selected to comprehensively cover the stages of deciduous and mixed dentition, extending to the consolidation of early permanent dentition. Regarding the handling of mixed-age studies, articles that included participants outside this range (e.g., older adolescents > 14 years or adults) were included only when data for the ≤14-year age group were reported separately. No restrictions were applied regarding publication year or language. Exclusion criteria comprised editorials, conference abstracts, and studies focusing exclusively on late adolescence (>14 years) or adulthood. Articles that did not meet these criteria were excluded, and any discrepancies during the selection process were resolved by consultation with a third reviewer.

### 2.4. Study Selection Process

Two previously trained independent reviewers (B.N.S.S. and D.C.F.) screened the identified records based on titles and abstracts, followed by full-text assessment. To ensure the reliability of the selection process, inter-reviewer agreement was quantified using Cohen’s Kappa coefficient (*K*). A pre-established threshold of *K* ≥ 0.81 was set to denote excellent agreement. The calculated value was *K* = 0.90, demonstrating a high level of consistency between the examiners. According to the study protocol, when evaluation of the title and abstract was insufficient to reach consensus, the full text was reviewed to resolve the disagreement. If discordance persisted after full-text screening, a third reviewer (L.R.P.) adjudicated the final decision.

### 2.5. Data Extraction from the Selected Studies

The articles selected from the database were exported to Rayyan (https://www.rayyan.ai/ (accessed on 31 May 2025); Rayyan, Cambridge, MA, USA; accessed on 31 May 2025) for individual screening and exclusion of studies unrelated to the established topic. Subsequently, the cleaned files extracted from WoS-CC were consolidated into a single dataset for analysis using the *bibliometrix* package in RStudio (version 4.5.0) [21,22]. The bibliometric variables extracted for analysis included article title, authorship, year of publication, journal name, Journal Impact Factor (JIF), total citation count, country of institutional affiliation, and keywords (both author-assigned and Keywords Plus^®^). Missing or incomplete entries (e.g., unidentified country of origin or missing publication year) were manually retrieved by cross-referencing digital object identifiers (DOIs) or the official journal websites.

### 2.6. Data Analysis

#### 2.6.1. Bibliometric Analysis

Bibliometric networks were developed using the Visualization of Similarities viewer (VOSviewer, version 1.6.20, Leiden, The Netherlands) [23] and the Biblioshiny software (version 5.0). *Bibliometrix* is an open-source R package designed to perform comprehensive scientific mapping analyses [24]. Its web-based interface, Biblioshiny, runs in R (version 4.3.3) and was used to visually analyze international collaboration networks, keyword co-occurrence, trending topics, and the thematic map.

Data related to connections between authorship and keywords were clustered to facilitate the understanding of findings. Terms associated with clusters and larger fonts had higher occurrences, while terms related to clusters and smaller fonts had lower occurrences. The lines indicate the connections between clusters, illustrating their relationships.

#### 2.6.2. Statistical Analysis

Spearman’s correlation test was used to assess the correlation among the number of citations, the journal impact factor, and the year of publication. Spearman’s correlation coefficient (rho) may be weak (rho > 0.30), moderate (rho > 0.50), high (rho > 0.70), or very high (rho > 0.9). The strength of correlation was interpreted according to the guidelines established by Hinkle et al. [25]. All analyses were performed in R software (Windows version 4.5) at a 5% significance level.

## 3. Results

The initial search yielded 281 articles. After applying the exclusion criteria, 107 WoS-CC articles published between 2006 and 2025 were included in the bibliometric analysis. Scientific production exhibited a mean annual growth rate of 7.57%, indicating a consistent increase in research interest over the analyzed period.

Regarding citation metrics, the documents received an average of 30.59 citations per article, with an annual mean of 2.89 citations per document, reflecting the relevance and dissemination of studies within the research field. Overall, 2358 cited references were identified within the dataset.

### 3.1. Keyword Analysis

**Figure 1:**
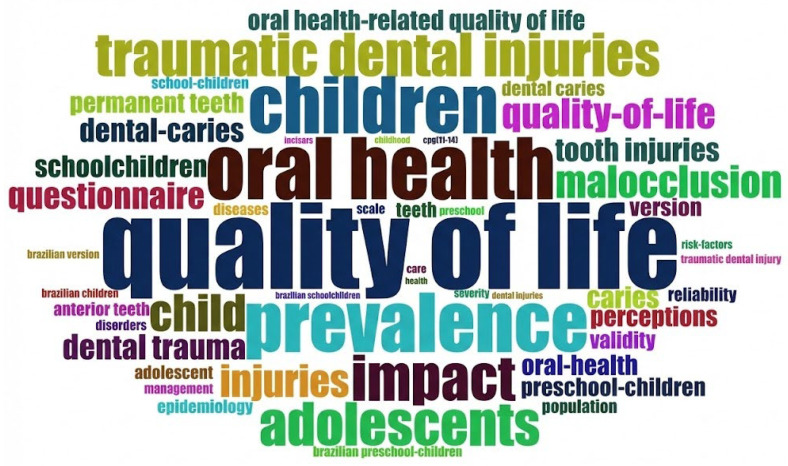
Word cloud showing the most frequently occurring keywords. The size of each term represents its total frequency across both Keywords Plus^®^ and author-assigned keyword lists.

**Figure 2:**
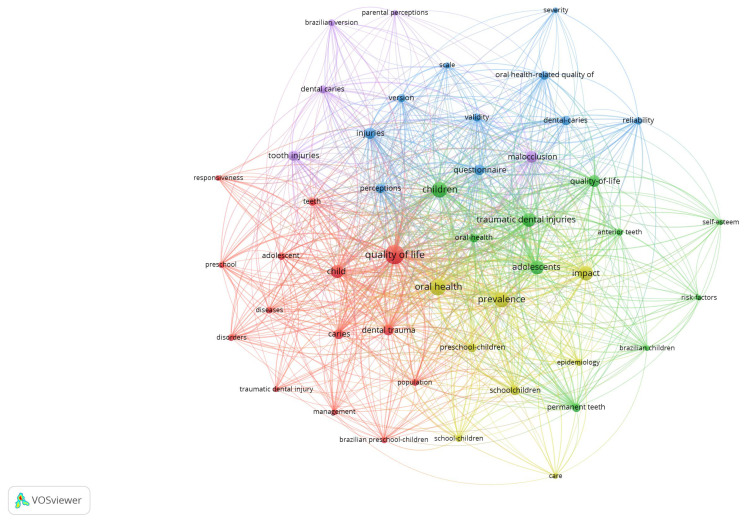
Network map of the most frequent keywords. Node colors represent keywords within the same cluster, indicating thematic proximity. Node sizes reflect the total number of occurrences across both Keywords Plus^®^ and author-assigned keyword lists. The lines (edges) between nodes represent the strength of co-occurrence.

The study identified 226 indexed keywords (Keywords Plus^®^) and 150 author-assigned keywords. As shown in Figure 1, the most frequently cited terms included “quality of life,” “prevalence,” “impact,” “dental trauma,” and “children.” Figure 2 displays the co-occurrence network map of these terms; node colors represent keyword clusters, while node size reflects the total number of occurrences across the analysis.

### 3.2. Publications and Citations over the Years

**Figure 3:**
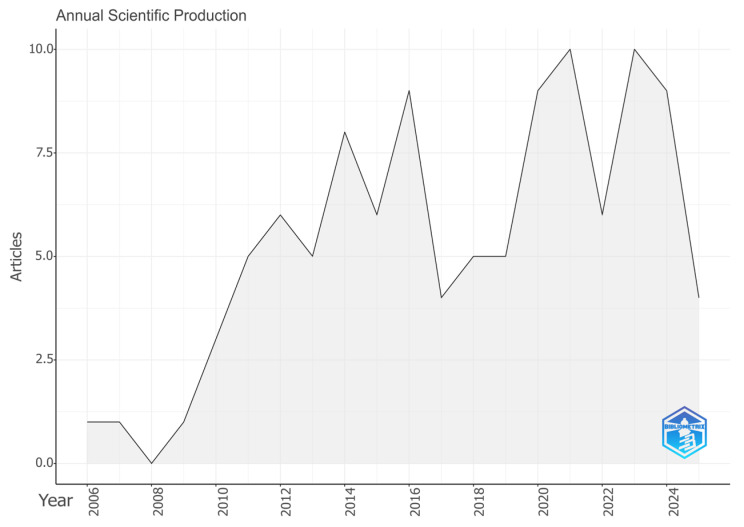
Average annual citations on the impact of dental trauma on children’s lives.

Figure 3 illustrates the annual average number of citations received by the included articles. Peaks in citation numbers were observed in 2013, 2017, and 2022.

### 3.3. Country Distribution

**Figure 4:**
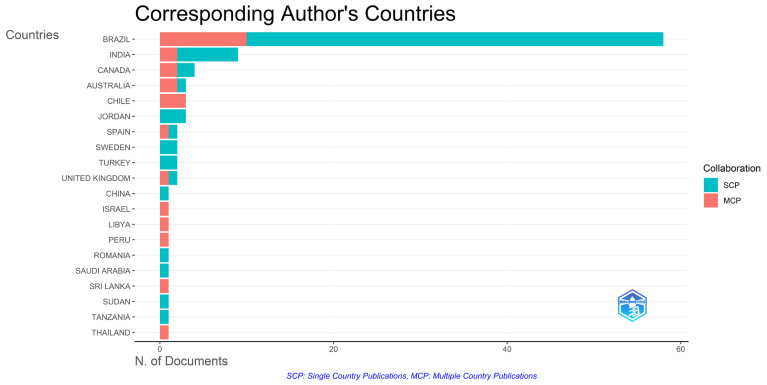
Distribution of corresponding authors by country and collaboration type. The bar chart represents the number of documents per country. Colors distinguish between single-country publications (SCP) and multiple-country publications (MCP), indicating the level of international collaboration.

The analysis of the countries of corresponding authors’ revealed that Brazil contributed the highest number of documents (*n =* 64), accounting for 60.4% of the total scientific output analyzed. Other countries with notable contributions included India (*n =* 9), Canada (*n =* 4), and Australia (*n =* 3) (Figure 4). Although contributions originated from multiple continents, most publications were authored by researchers based within national research networks.

**Figure 5:**
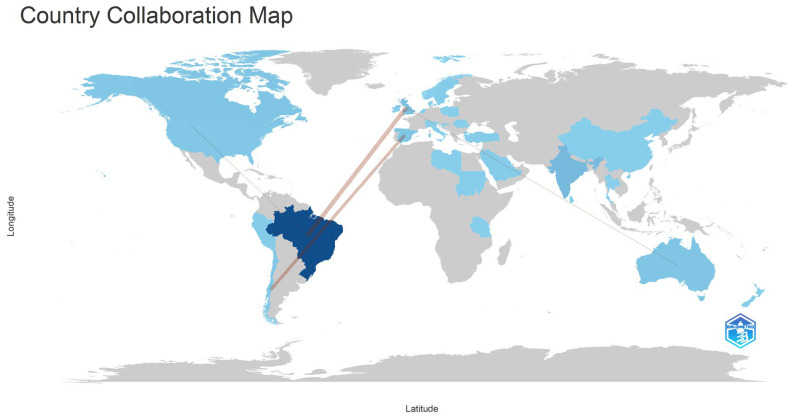
Global collaboration map of research on dental trauma and children’s quality of life. Color intensity reflects the volume of publications per country, while the lines represent co-authorship links between different countries.

Countries involved in this research area are highlighted in blue, with darker shades indicating higher publication output. The lines connecting countries represent collaborative links between them. Brazil emerges as the main hub of scientific production in this field, showing the highest concentration of publications, as indicated by the darkest blue shading (Figure 5).

### 3.4. Authors’ Contributions

A total of 381 authors contributed to the included articles, with an average of 0.3 documents per author and 5.1 co-authors per article. The international co-authorship index was 25.2%.

**Figure 6:**
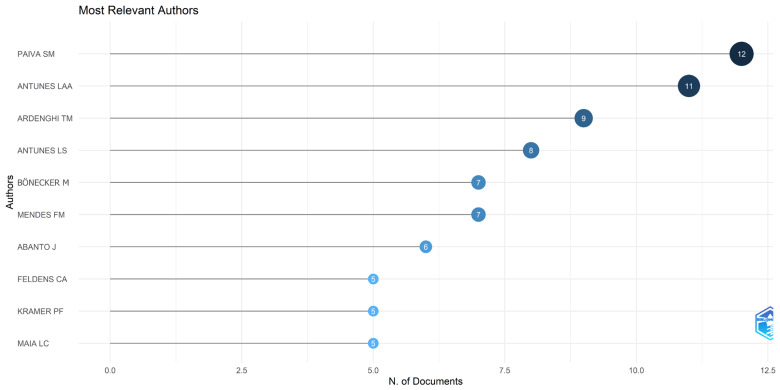
Most relevant authors based on publication count. The lollipop chart illustrates the total number of documents published by each author. The prominent positions of authors such as Paiva SM and Antunes LAA highlight the substantial contribution of specific research groups to the global literature on dental trauma.

Analysis of the most productive authors identified Paiva SM had the highest number of publications (*n =* 12), followed by Antunes LAA (*n =* 11), Ardenghi TM (*n =* 9), Antunes LS (*n =* 8), Bönecker M (*n =* 7), and Mendes FM (*n =* 7) (Figure 6). These authors represent key contributors to the development of scientific knowledge on dental trauma in children, particularly within the Brazilian research context, which accounts for a substantial share of the academic output in this field.

When adjusting for fractionalized production (which distributes authorship credit proportionally among co-authors), the highest values were observed for Antunes LAA (2.38), Paiva SM (2.05), Ardenghi TM (1.81), Antunes LS (1.59), Rajab LD (1.50), and Bönecker M (1.34). These findings indicate that, beyond absolute publication counts, the cited authors demonstrate substantial and recurrent involvement in collaborative scientific research.

### 3.5. Journals and Articles

**Figure 7:**
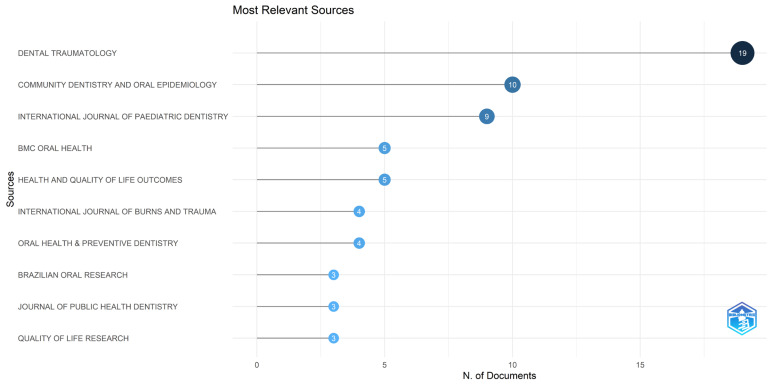
Most relevant journals based on the number of publications. The lollipop chart ranks the sources according to the total number of documents published on the impact of dental trauma on the quality of life of children and adolescents.

The analyzed scientific production is distributed across several specialized journals (Figure 7). *Dental Traumatology* was the most prolific source, with 19 publications. Other journals with notable contributions included *Community Dentistry and Oral Epidemiology* (*n =* 10) and the *International Journal of Paediatric Dentistry* (*n =* 9). *BMC Oral Health* and *Health and Quality of Life Outcomes* each published five articles on the topic, while the *International Journal of Burns and Trauma* and *Oral Health & Preventive Dentistry* contributed four publications each.

**Figure 8:**
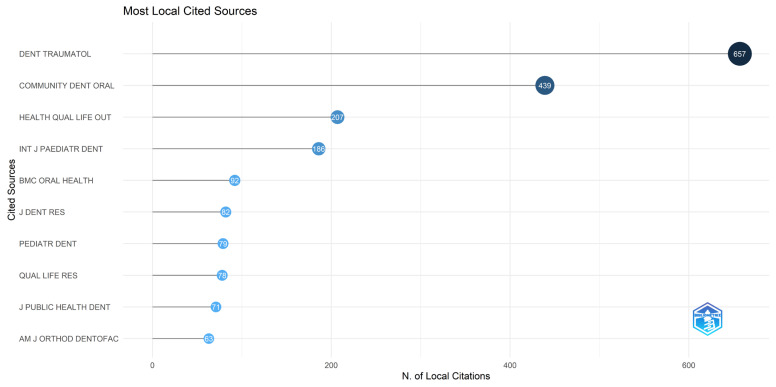
Most locally cited journals within the research sample. The chart illustrates the journals that are most frequently referenced by the articles included in this bibliometric study, reflecting the core literature that supports research on dental trauma and children’s quality of life.

Regarding citation impact (Figure 8), *Dental Traumatology* was the most referenced source, with 657 citations, followed by *Community Dentistry and Oral Epidemiology* (439 citations), *Health and Quality of Life Outcomes* (207 citations), and the *International Journal of Paediatric Dentistry* (186 citations). Other notable sources within the citation network included *BMC Oral Health* (*n =* 92), the *Journal of Dental Research* (*n =* 82), and *Pediatric Dentistry* (*n =* 79).

**Figure 9:**
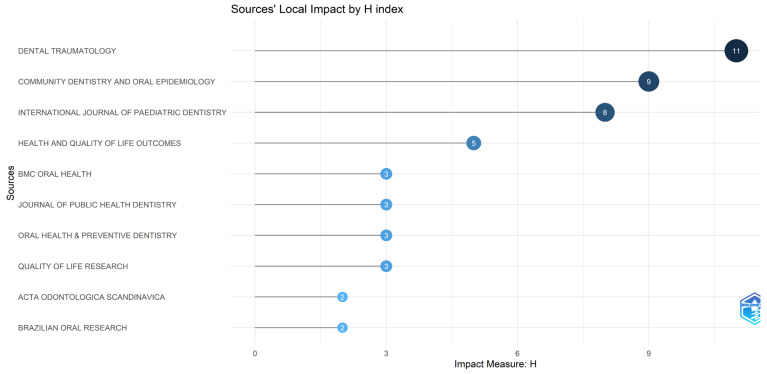
Local impact of journals based on the H-index. The lollipop chart ranks the sources according to their H-index, reflecting both the number of published articles and their citation impact regarding dental trauma and its influence on children’s quality of life.

The local impact measured by the H-index is presented in Figure 9. This indicator simultaneously evaluates productivity and citation relevance within the analyzed dataset. Consistent with previous metrics, *Dental Traumatology* exhibited the highest local impact (H = 11), followed by *Community Dentistry and Oral Epidemiology* (H = 9) and the *International Journal of Paediatric Dentistry* (H = 8). Other journals contributing to the core literature in the field included *Health and Quality of Life Outcomes* (H = 5), as well as *BMC Oral Health*, the *Journal of Public Health Dentistry*, and *Oral Health & Preventive Dentistry*, each with an H-index of 3 (Figure 9).

The article by Abanto J et al. [23], published in *Community Dentistry and Oral Epidemiology*, received the highest number of citations (*n =* 270), with an annual average of 18 citations. This was followed by the study by Lam R [24], published in the *Australian Dental Journal*, which accrued 258 citations and exhibited the highest annual citation rate (25.8 citations/year). Spearman’s correlation analysis revealed a weak positive correlation between citation counts and journal impact factor (rho = 0.37, *p* < 0.001), as well as a strong negative correlation between citation counts and year of publication (rho = −0.84, *p* < 0.001).

### 3.6. Trending Topics and Thematic Map

**Figure 10:**
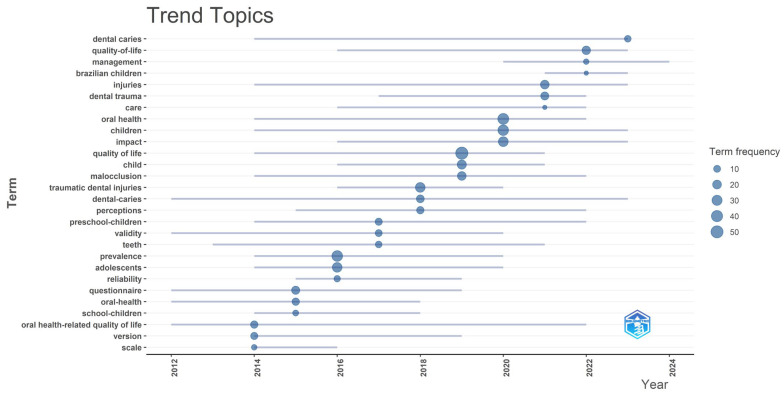
Evolution of trending topics based on author keywords (2012–2024). The chart displays the frequency and temporal distribution of keywords, where circle size represents term frequency.

The analysis of trending topics illustrates the frequency of the most recurrent themes in the literature addressing the impact of dental trauma on children over time. The occurrence of terms such as “dental trauma,” “quality of life,” “oral health,” “children,” and “management” has increased since 2018 (Figure 10).

**Figure 11:**
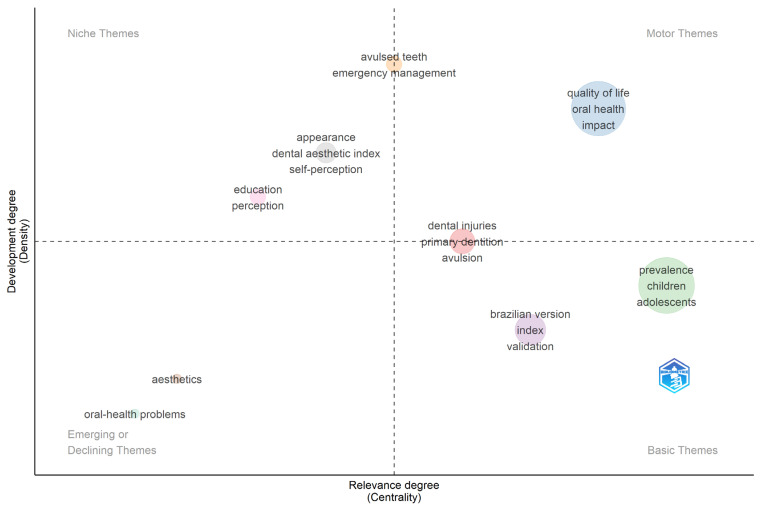
Key motor, basic, emerging/declining, and niche themes identified from the authors’ keywords. The thematic map categorizes keywords into four quadrants based on their centrality (relevance) and density (development degree).

Figure 11 presents topics related to the impact of dental trauma on children’s lives, organized into four quadrants according to their centrality (relevance and connections with other themes) and density (level of thematic development).

The topics “quality of life” and “impact on oral health” were positioned in the motor theme quadrant (high centrality and density). Conversely, terms such as “aesthetics” and “oral health problems” appeared in the quadrant associated with emerging or declining themes (low density and centrality). Topics such as “avulsed” and “emergency management” were characterized as niche themes (high density, low centrality).

## 4. Discussion

This study provides a bibliometric overview of the scientific literature addressing the impact of dental trauma on children’s lives, with emphasis on OHRQoL, by examining publication patterns, citation metrics, collaboration networks, and thematic trends. By applying established bibliometric techniques to studies indexed in the Web of Science Core Collection (WoS-CC)—a database recognized for its rigorous selection criteria and widespread use in bibliometric research [19]—this analysis offers a structured perspective on the evolution of knowledge in this field over time.

The corpus comprised 107 articles published between 2006 and 2025, revealing a relatively stable publication pattern characterized by a low annual growth rate. Rather than indicating stagnation, this stability may reflect the consolidation of consistent conceptual frameworks and methodological approaches within the field. At the same time, the lack of sustained growth in publication volume suggests that, despite the clinical and public health relevance of dental trauma in childhood, this topic has not undergone substantial expansion in recent years. This pattern highlights opportunities for diversification of research designs and analytical perspectives, particularly through the incorporation of psychosocial dimensions, social inequalities, and emerging tools such as digital health resources and artificial intelligence.

The comprehensive search used in this study included terms related to biopsychosocial and educational factors that could act as possible confounding factors between traumatic dental injuries and OHRQoL. However, many of these domains appeared superficially among the keywords or as themes in the articles identified in this bibliometric review. This finding likely indicates a substantive gap in the existing body of evidence, suggesting that the impact of dental trauma on children’s educational experiences and broader psychosocial development remains underexplored.

Although quantitative growth was limited, citation indicators point to a field with considerable scientific influence. The mean citation rate of 31.83 citations per document and an annual average of approximately two citations per article indicate sustained academic interest and relevance, particularly within paediatric dentistry, epidemiology, and public health contexts. In addition, the average document age of 7.1 years suggests that influential publications are relatively recent, supporting the interpretation that the literature remains active and continues to inform current research and practice. The diversity of keywords further reinforces this interpretation, revealing a wide range of approaches and subtopics, including quality of life assessment, family perceptions, epidemiological analyses, and social determinants of health.

Collaboration patterns constitute another important dimension of this bibliometric profile. The predominance of multi-authored publications, with an average of 5.1 authors per article and only two single-authored papers, indicates a collaborative and multidisciplinary research environment. However, the international co-authorship rate of 25.2% remains modest. Given that dental trauma is strongly influenced by geographic, cultural, and socioeconomic contexts [8,9], limited international collaboration may constrain the comparability of findings across different settings. Expanding cross-national partnerships could therefore strengthen the external validity of future studies and promote more comprehensive interpretations of trauma-related impacts on children’s lives.

The analysis of the most cited articles underscores the prominence of OHRQoL as a central outcome in research on childhood dental trauma. Highly cited studies consistently demonstrate associations between traumatic dental injuries and impairments in self-esteem, social interaction, and overall well-being [26,27,28,29,30]. The presence of journals such as *Health and Quality of Life Outcomes* and *Quality of Life Research* among the most influential outlets highlights the interdisciplinary nature of this research area, bridging dentistry with psychology, public health, and the social sciences.

A notable finding is the predominance of Brazilian authors and institutions among the most cited contributions, including Gomes MC [28], Piovesan C [29], and Kramer PF [30]. While this pattern reflects Brazil’s strong engagement in investigating the consequences of dental trauma—often within socially vulnerable populations—it also suggests a regional concentration of knowledge production. Such overrepresentation may be partially related to national research priorities, funding structures, and the widespread application of validated OHRQoL instruments in Brazilian paediatric populations. At the same time, this finding underscores the importance of encouraging broader geographic participation to balance perspectives and reduce potential regional bias in the literature.

Citation patterns further suggest that studies integrating clinical data on dental trauma with validated OHRQoL instruments tend to achieve greater scientific visibility. This finding reinforces the relevance of patient- and family-centered outcomes in paediatric dentistry and emphasizes that the impact of trauma extends beyond clinical signs to encompass emotional and social dimensions of child development. From a bibliometric perspective, these patterns indicate that OHRQoL assessment constitutes a thematic nucleus around which influential research in this field has been structured.

Another pattern of citation counts identified in this bibliometric analysis is the weak correlation with the journal’s impact factor and the high correlation with the year of publication. The weak positive association between citation counts and journal impact factor suggests that, although higher-impact journals may offer greater visibility, citation performance is also influenced by thematic relevance and methodological robustness. On the other hand, the strong negative correlation between citations and year of publication reflects a well-established citation maturation effect, whereby older publications have had more time to accumulate citations.

With respect to thematic analysis, the results indicate that while topics such as the prevalence and clinical impact of dental trauma are well established, they are more frequently examined in relation to older children and adolescents. In contrast, themes directly addressing preschool-aged children—particularly the specific implications of dental trauma for early childhood development and quality of life—remain less central and more fragmented. This pattern suggests that, although preschool-aged children are included within the broader paediatric literature, their experiences are often underrepresented or treated as secondary outcomes. The present study addresses this gap by emphasizing the need for more focused and integrated investigations targeting early childhood populations.

In this regard, the thematic map elucidates critical gaps by highlighting that issues specific to early childhood are not yet positioned among the field’s central motor themes. While broad constructs such as “quality of life” appear as well-established and highly connected topics, themes more specifically associated with preschool-aged children—such as esthetic concerns, functional limitations, and family-related outcomes—remain peripheral or weakly interconnected. This distribution suggests that future investigations would benefit from moving beyond descriptive prevalence estimates to adopt more integrative perspectives that consider specific developmental stages. Prioritizing these themes is essential to support the development of a more balanced and age-sensitive research agenda in pediatric dental trauma.

Overall, the bibliometric indicators suggest a field characterized by consistent scientific production, moderate international visibility, and a strong thematic emphasis on quality of life outcomes. Rather than indicating full scientific consolidation, these findings point to a phase of relative stability accompanied by clear opportunities for conceptual renewal and expansion. Future research directions discussed in the literature—such as the inclusion of socioeconomic, cultural, and gender-related variables, as well as qualitative and mixed-methods approaches—are aligned with the thematic gaps identified through keyword and citation analyses.

Finally, the limited level of international collaboration observed—particularly among leading contributor countries such as Brazil—highlights an important opportunity to strengthen partnerships with other regions, especially within the Global South. Such collaborations could broaden the contextual understanding of dental trauma, support the development of culturally sensitive preventive strategies, and inform public health policies tailored to diverse childhood populations [31]. At the same time, these collaboration patterns should be interpreted considering the methodological boundaries inherent to bibliometric analyses, which may influence the visibility and representation of specific countries, research groups, and thematic emphases within the literature.

This study has some limitations. The exclusive use of the Web of Science Core Collection (WoS-CC) may have introduced selection bias, as relevant studies indexed in other databases may not have been captured. Although no language restrictions were applied, language and citation biases inherent to bibliometric analyses cannot be excluded, particularly those favoring older and English-language publications. In addition, the methodological quality of the included studies was not assessed; therefore, the findings should be interpreted as indicators of scientific production and thematic structure rather than as an evaluation of clinical evidence. Furthermore, bibliometric indicators were not normalized by population size, Gross Domestic Product (GDP), or number of researchers; as a result, countries with strong research traditions in paediatric dentistry (Brazil and India) may appear disproportionately represented in absolute publication counts. Finally, the statistical analyses employed were descriptive in nature, and future studies using larger datasets may benefit from more advanced analytical approaches.

## 5. Conclusions

This bibliometric analysis clarifies how research on the impact of dental trauma on children’s oral health-related quality of life has evolved in terms of productivity, thematic focus, and collaboration patterns. Rather than indicating expansion, the mapped indicators point to thematic stability alongside limited international integration, highlighting opportunities for diversification of perspectives and methodological approaches. From a healthcare and policy standpoint, these findings help identify underexplored areas and inform future research agendas aimed at strengthening the evidence base to support preventive strategies, child-centered care, and informed decision-making in paediatric oral health.

## Figures and Tables

**Table 1:** Search strategy used in the Web of Science Core Collection database.

Conceptual Block	Search Terms Used
#1: Dental Trauma	“Dental Trauma” OR “Tooth Trauma” OR “Traumatic Dental Injuries”
#2: Target Population	“Child” OR “Children” OR “Child, Preschool” OR “Children, Preschool” OR “Preschool child” OR “Preschool Children” OR “Schoolchildren”
#3: Biopsychosocial and Educational Outcomes	“Models, Biopsychosocial” OR “Biopsychosocial Model” OR “Bullying” OR “Absenteeism” OR “Quality of Life” OR “Oral Health Related Quality of Life” OR “Self Concept” OR “Self Image” OR “Self Perception” OR “Self Esteem” OR “Academic Achievement” OR “Academic Performance” OR “School Performance” OR “Educational Measurement” OR “Educational Test Score”
Final Query	#1 AND #2 AND #3

**Source**: Developed by the authors.

## Data Availability

No new data were created or analyzed in this study. Data sharing is not applicable to this article.

## Abbreviations

The following abbreviations are used in this manuscript:

OHRQoL	Oral health-related quality of life
WoS-CC	Web of Science
